# A Three-Pillar Approach to Laboratory Sustainability in Environmental Analysis

**DOI:** 10.1021/acssusresmgt.5c00425

**Published:** 2025-09-20

**Authors:** Helena Rapp-Wright, Caroline Pollard

**Affiliations:** † 4957 MRC Centre for Environment and Health, Environmental Research Group, School of Public Health, Imperial College London, 86 Wood Lane, London W12 0BZ, United Kingdom; ‡ Leverhulme Research Centre for Forensic Science, University of Dundee, Small’s Wynd, Dundee DD1 4HN, United Kingdom

**Keywords:** Sustainable Development Goals (SDGs), green laboratory, sustainability stool framework, social equity, economic sustainability

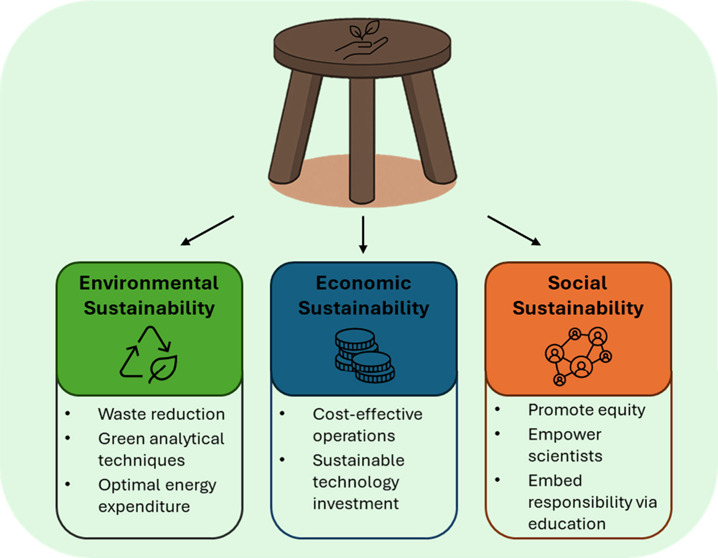

The United Nations Sustainable Development Goals (SDGs) call on everyone to support sustainability to address global environmental challenges.^1^ As the urgency of this matter intensifies, the scientific community faces increasing pressure to align common research practices with sustainability frameworks.^2^ Environmental analysis contributes significantly to monitoring pollutants, assessing ecosystem health, and informing regulatory policies.^3^ However, the very processes designed to protect the environment often generate substantial waste, consume large amounts of energy, and rely heavily on hazardous chemicals and disposable materials.^4–6^ Balancing the demand for high-quality, reproducible data with the responsibility to reduce environmental impact is an increasingly urgent priority. Both academia and industry are facing pressure to consider the sustainability of their work. This challenge has prompted the development of greener analytical methods and assessment tools to guide more responsible laboratory practices.^4^ Despite progress in reducing resource use and waste, a broader, systems-based approach is essential for long-term sustainability beyond the laboratory.

To address this, a three-pillar approach, often termed the “sustainability stool”, provides a framework to integrate environmental, economic, and social considerations into every stage of method development and laboratory decision-making (Figure 1).^7^ Like a three-legged stool, all three components must be equally considered to support truly sustainable practices. Focusing on only environmental metrics (e.g., solvent reduction or waste minimization) without accounting for economic feasibility or social usability can hinder the adoption of sustainable methods.^8^ For example, greener alternatives can be environmentally ideal but are often unsustainable due to high costs, more expensive instrumentation, or extensive staff retraining; thus, they may not be economically or socially sustainable.

**Figure 1:**
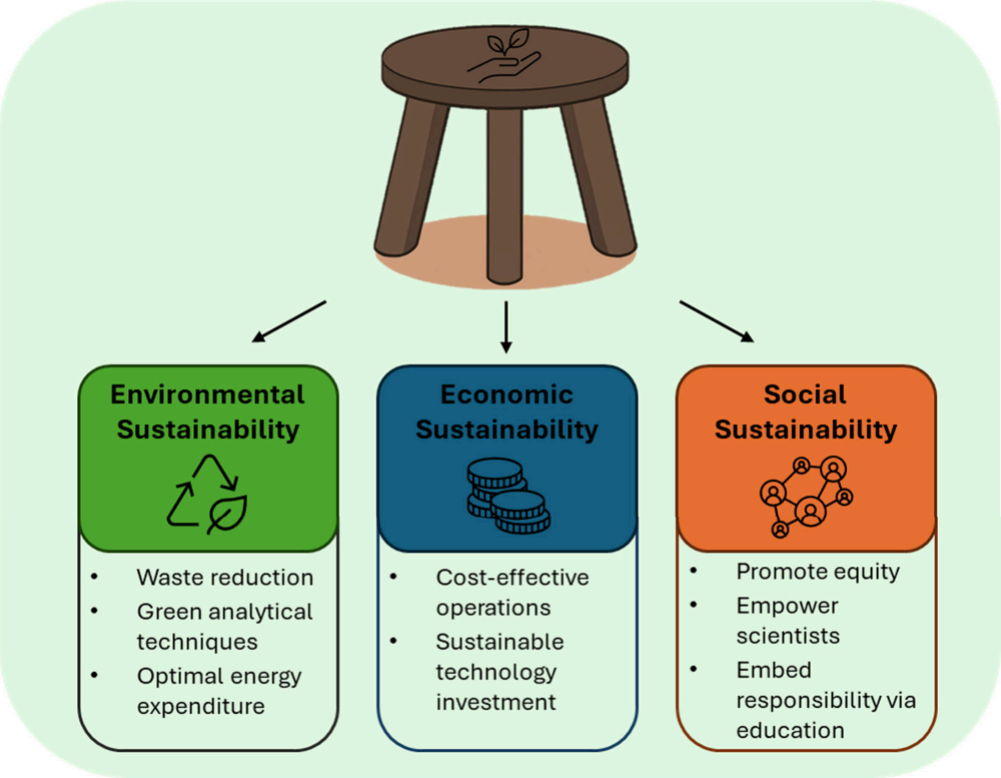
“Sustainability stool” framework illustrating the three interdependent pillars of sustainable scientific practice: environmental, economic, and social sustainability. Each pillar supports a balanced approach to responsible research in environmental analysis.

## Environmental Sustainability

This component focuses on minimizing the carbon footprint of laboratory work following the introduction of the “12 Principles of Green Chemistry”.^9^ In 2013, the original principles were adapted to produce seven principles specific to green analytical chemistry,^10,11^ followed by the presentation of the “10 Principles of green sample preparation” in 2022.^12^ This has encouraged scientists to use sustainability frameworks, accreditation-like schemes (e.g., The Laboratory Efficiency Assessment Framework (LEAF), The Laboratory Efficiency Action Network (LEAN), and My Green Lab), and “greenness” metrics such as the Analytical GREEnness calculator (AGREE), which can help researchers assess the ecological impact of their workflows and identify opportunities for improvement.^4,6,13,14^ This includes reducing hazardous solvent use, minimizing single-use plastics, optimizing energy consumption, and managing chemical waste responsibly. For example, switching to bio-based solvents or reusing glassware where feasible can significantly reduce waste and emissions. Method optimization, such as reducing chromatographic run times or using greener instrumentation like supercritical fluid chromatography (SFC), also decreases energy and solvent use.^15^ While high-quality data are essential, integrating environmental considerations ensures that laboratory practices do not contradict the very purpose of environmental protection.

## Economic Sustainability

Economic sustainability in environmental analysis emphasizes the efficient use of resources without incurring unsustainable financial burdens in ensuring the long-term sustainability of the research landscape. As laboratories typically operate on tight budgets, cost-effectiveness becomes a paramount concern when developing new methods. Economically sustainable approaches include reducing consumables, extending instrument life spans through maintenance rather than replacement, and selecting cost-effective reagents or materials. Furthermore, Design of Experiments (DoE) instead of the traditional one-factor-at-a-time (OFAT) process can minimize the number of trials needed for method validation, saving both time and materials, and therefore money.^16^ *In silico* simulations and computer-assisted method development can also be used as a rapid and greener technique.^17^ More importantly, investment in sustainable technologies, such as energy-efficient equipment or automation, can offer long-term cost savings. By weighing economic impacts alongside environmental goals, the sustainability stool helps ensure that greener practices are not only environmentally responsible but also financially viable and scalable. This ensures job security for staff, plus a work environment that can invest in sustainable research.

A different aspect of economic sustainability is the fair distribution of resources. For example, the repair of faulty equipment is often the preferred and/or only option in low-income countries. This highlights the responsibility for well-funded laboratories to share or redistribute equipment, feeding also into the environmental and social legs of the stool.

## Social Sustainability

This final pillar considers the human element involved in scientific research, which encompasses both the scientist and the scientific community. It is important to create safe and inclusive working environments, ensuring proper training on sustainable practices as well as social equality and accessibility to greener technologies for everyone.^18^ Promoting sustainability literacy and embedding responsible lab practices into education and standard operating procedures empower scientists to make informed decisions.

Analytical methods should be not only technically rigorous but also practical for users across different regions and levels of expertise. For instance, overly complex or resource-intensive methods may exclude underfunded labs, hindering global collaboration and data comparison.^19,20^ The social pillar reinforces that sustainability must be centred on people, supporting equity, safety, and knowledge sharing within the scientific community.

## Conclusions

In summary, the stool highlights the need to balance sustainable practices across the three pillars. Economic sustainability remains the most influential factor in terms of the uptake of sustainable practices, as economic viability is often the driving factor in both academia and industry. Nevertheless, by adopting the sustainability stool framework for environmental analysis, laboratories can develop solutions that are environmentally responsible, socially adoptable, and economically feasible. This holistic approach provides a tool that can be implemented by all environmental scientists to achieve the SDGs.
